# Association of schistosome infection with adiposity in Tanzania

**DOI:** 10.3389/fpubh.2022.1008101

**Published:** 2023-01-04

**Authors:** Khanh Pham, George PrayGod, Daniel Faurholt-Jepsen, Mette F. Olsen, Bazil Kavishe, Brenda Kitilya, Paul L. A. M. Corstjens, Claudia J. de Dood, Henrik Friis, Suzanne Filteau, Jennifer A. Downs, Robert N. Peck

**Affiliations:** ^1^Division of Infectious Diseases, Weill Cornell Medicine, New York, NY, United States; ^2^Mwanza Research Centre, National Institute for Medical Research, Mwanza, Tanzania; ^3^Department of Infectious Diseases, Rigshospitalet, Copenhagen, Denmark; ^4^Department of Nutrition, Exercise and Sports, University of Copenhagen, Copenhagen, Denmark; ^5^Department of Cell and Chemical Biology, Leiden University Medical Center, Leiden, Netherlands; ^6^Faculty of Epidemiology and Population Health, London School of Hygiene and Tropical Medicine, London, United Kingdom; ^7^Center for Global Health, Weill Cornell Medical College, New York, NY, United States; ^8^Department of Medicine, Weill Bugando School of Medicine, Mwanza, Tanzania

**Keywords:** schistosome infection, adiposity, HIV, antiretroviral therapy, cardiovascular disease

## Abstract

**Background:**

Observational studies in humans have reported a link between schistosome infection and lower adiposity, but this may be explained by socioeconomic and demographic factors, intensity of infection, or common co-infections such as HIV.

**Methods:**

This was a cross-sectional study that investigated the relationship between schistosome infection and adiposity in a large, well-described cohort of Tanzanian adults living with and without HIV. Cross-sectional data were collected among adults living in Mwanza, Tanzania who were enrolled in the Chronic Infections, Co-morbidities and Diabetes in Africa (CICADA) cohort study. Schistosome circulating anodic antigen, secreted by both *Schistosoma mansoni* and *haematobium* which are endemic to Tanzania, was quantified from stored samples. Schistosome infection diagnosed by serum circulating anodic antigen levels. The primary outcome was fat mass measured by bioimpedance analysis. Secondary outcomes included fat-free mass, waist circumference, mid-upper arm circumference, and body mass index.

**Results:**

The study enrolled 1,947 adults, of whom 1,923 (98.8%) had serum available for schistosome testing. Of these, 873 (45.4%) had a serum circulating anodic antigen ≥30 pg/mL, indicating schistosome infection. Compared to uninfected individuals, those with schistosome infections had −1.1 kg [95% CI −1.9 to −0.3] lower fat mass after adjusting for age, sex, physical activity, tobacco use, education level, and socioeconomic status. Infected participants also had lower waist circumference, mid-upper arm circumference, and body mass index. Fat-free mass was not different between the two groups. Neither being HIV-infected, nor receiving antiretroviral therapy, modified associations between schistosome infection and adiposity. These associations were also not affected by Schistosoma worm burden.

**Conclusions:**

Schistosome infection was associated with lower fat mass and less central adiposity without a difference in muscle mass, irrespective of confounders, HIV status, or the intensity of schistosome infection. Future studies should adjust for socioeconomic and demographic factors that are associated with schistosome infection and adiposity. Identifying mechanistic pathways by which schistosome infection reduces adiposity while preserving muscle mass could yield new strategies for obesity control and cardiovascular disease prevention.

## Introduction

*Schistosoma* parasites are water-borne helminths that affect over 200 million people worldwide and infection is highly prevalent in fishing communities near Lake Victoria in Tanzania (1, 2). Schistosome infection disproportionately affects the poor, particularly those with limited access to clean water, as individuals become infected when they have physical contact with contaminated water. Once the parasites penetrate through human skin, adult worms lay eggs in the host venules that migrate through host tissue and cause significant, species-dependent morbidity and mortality in various organ systems (1).

Although most research has focused on the deleterious health effects of schistosomes, a few studies have reported beneficial effects of schistosome infection on common measures of obesity, such as body mass index (BMI) (3, 4). However, these studies have not examined whether this reduction in BMI in schistosome-infected adults represents a true reduction in fat mass (FM), they have not adjusted for confounding socioeconomic and demographic factors associated with schistosome infection, and they have not yet explored whether the effect of schistosomes on obesity might be modified by the intensity of infection or common co-infections such as HIV. Given the distinct socio-ecological characteristics of schistosome infection and obesity in Africa, robust assessment of factors associated with schistosome (5, 6) infection, adiposity, or both is essential. A clearer analysis would more accurately characterize the relationship between schistosome infection and adiposity, and possibly lead toward understanding underlying mechanisms.

In this novel study, we hypothesized that schistosome-infected participants would have lower FM than schistosome-uninfected participants, regardless of HIV or antiretroviral therapy (ART) status or intensity of schistosome infection, even after adjusting for significant socioeconomic and demographic factors. To test these hypotheses, we quantified associations between cross-sectional measures of adiposity, including FM, fat-free mass (FFM), waist circumference (WC), mid-upper arm circumference (MUAC), and BMI, while adjusting for characteristics associated with both schistosome infection and adiposity. We also examined the effect modification by HIV and ART.

## Methods

### Study design, setting, and laboratory testing

We analyzed cross-sectional data collected between October 2016 and November 2017 from adults (age ≥18 years) living in Tanzania who were enrolled in the Chronic Infections, Co-morbidities and Diabetes in Africa (CICADA) study. CICADA examined the risk factors and burden of non-communicable diseases in three groups of participants (HIV-infected, HIV-infected on ART, and HIV-uninfected) and was registered at https://clinicaltrials.gov (NCT03106480). Further details were published previously (7, 8).

Schistosome circulating anodic antigen (CAA), secreted by both *Schistosoma mansoni* and *haematobium*, was quantified in stored serum samples using a lateral flow assay (9). Both species are endemic to Tanzania, with *S. mansoni* predominant. Schistosome infection was defined as CAA ≥30 pg/mL.

### Anthropometry and body composition

Participants underwent bioimpedance analysis to calculate FM and FFM using a body composition analyzer (Tanita BC418, Japan). Weight, height, MUAC, and waist and hip circumferences were determined as previously described (7, 8). Physical activity was computed from the WHO STEPS questionnaire and reported in minutes/day (10). Demographic information was collected; socioeconomic status (SES) was calculated using principal component analysis (11).

### Ethics statement

Permission to conduct this study was granted by the National Institute for Medical Research in Tanzania and Weill Cornell Medicine in New York. Participants provided written informed consent in Kiswahili.

### Data management and statistics

Data were collected using CSPro and analyzed in Stata MP/Version 17 (College Station, Texas). We applied descriptive statistics, using means and standard deviations (SD) for continuous variables and frequencies and percentages for categorical variables. The primary outcome was FM, and secondary outcomes included other measures of adiposity. We used linear and logistic regression to compare characteristics between schistosome-infected and uninfected individuals and measure the effect of schistosome infection intensity on adiposity. We then performed adjusted regression models, accounting for age, sex, physical activity, tobacco use, education level, and SES. We used interaction terms to assess for effect modification by HIV and ART on schistosome infection and adiposity. *P*-values <0.05 were considered significant.

## Results

Among 1,923 participants included, 873 (45.4%) were considered schistosome-infected. The schistosome-infected group was ~1.5 years younger and had a lower frequency of females (Table 1). Distributions of HIV-infected ART naïve, HIV-infected on ART, and HIV-uninfected participants were similar between those with and without schistosome infection. Tuberculosis and malaria infection status and serum C-reactive protein levels were also similar between groups.

**Table 1:** Sociodemographic and other characteristics in schistosome-infected vs. uninfected individuals.

**Characteristic**	**Schistosome-infected** **(*n* = 873) mean (SD) or number (%)**	**Schistosome-uninfected** **(*n* = 1,050) mean (SD) or number (%)**	***p*** **-value**
Age in years	39.9 (11.4)	41.3 (12.2)	**0.012**
Female sex	477 (54.6%)	661 (63.0%)	**< 0.001**
HIV-infected on ART	135 (15.5%)	189 (18.0%)	0.14
HIV-infected not on ART	443 (50.7%)	506 (48.2%)	0.27
HIV-uninfected	295 (33.8%)	355 (33.8%)	0.99
Current malaria infection	21 (2.4%)	17 (1.6%)	0.22
Current tuberculosis disease	10 (1.2%)	8 (0.8%)	0.39
C-reactive protein (mg/L)	13.9 (34.5)	11.7 (32.1)	0.16
Physical activity (time spent per day walking or cycling for travel/work) (min)	84.2 (86.3)	75.4 (75.4)	**0.037**
Have you ever smoked any tobacco?^a^			**< 0.001**
Yes	245 (28.1%)	220 (20.9%)	
No	627 (71.8%)	826 (78.7%)	
Have you ever drunk any alcohol?^a^			0.10
Yes	638 (73.1%)	730 (69.5%)	
No	234 (26.8%)	316 (30.1%)	
Education level^a^			**0.026**
- No formal education	157 (18.0%)	158 (15.0%)	
- Primary	597 (68.4%)	712 (67.8%)	
- Secondary or tertiary	100 (11.5%)	153 (14.6%)	
- Vocational	16 (1.8%)	25 (2.4%)	
Socioeconomic status^a^			**< 0.001**
- Lower tertile	338 (38.7%)	301 (28.7%)	
- Middle tertile	313 (35.9%)	327 (31.1%)	
- Upper tertile	219 (25.1%)	420 (40.0%)	

a Numbers do not add up due to missing data.

The bold values indicate statistical significance, *p*-value < 0.05.

Schistosome-infected persons had lower education level and SES, more physical activity, and more tobacco use than their uninfected counterparts, suggesting that these were potential confounders.

Schistosome-infected individuals had significantly lower FM than uninfected individuals (unadjusted regression coefficient: −2.9 kg [−3.8 to −2.1]; *p* < 0.001, Table 2), as well as lower WC, MUAC, and BMI. Schistosome-infected individuals more frequently had underweight and normal-weight BMI. FFM was not different between those with and without schistosome infection.

**Table 2:** Measures of adiposity and body composition in schistosome-infected vs. uninfected individuals.

**Characteristic**	**Schistosome-infected (*n* = 873) mean (SD) or number (%)**	**Schistosome-uninfected (*n* = 1,050) mean (SD) or number (%)**	**Unadjusted (coefficient [95% CI], *p*-value)**	**Adjusted^a^ (coefficient [95% CI], *p*-value)**
Primary outcome				
Fat mass (kg)	12.0 (8.3)	14.9 (10.0)	−2.9 [−3.8, −2.1], ***p*** **< 0.001**	−1.1 [−1.9, −0.3], ***p*** **= 0.009**
Secondary outcomes				
Fat free mass (kg)	44.8 (7.5)	44.5 (7.7)	0.3 [−0.4, 1.0], *p* = 0.45	−0.4 [−0.9, 0.2], *p* = 0.23
Waist circumference (cm)	77.7 (9.7)	81.0 (11.9)	−3.3 [−4.3, −2.3], ***p*** **< 0.001**	−1.6 [−2.7, −0.6], ***p*** **= 0.003**
Mid-upper arm circumference (cm)	26.3 (3.7)	27.3 (4.4)	−1.0 [−1.4, −0.6], ***p*** **< 0.001**	−0.4 [−0.8, −0.03], ***p*** **= 0.037**
Body mass index (kg/m^2^)	21.2 (4.0)	22.4 (4.8)	1.2 [−1.6, −0.8], ***p*** **< 0.001**	−0.5 [−1.0, −0.09], ***p*** **= 0.018**
Body mass index categories^bc^:			−0.5 [−0.6, −0.3], ***p*** **< 0.001**	−0.2 [−0.4, 0.02], *p* = 0.078
Underweight (<18.5 kg/m^2^)	212 (24.3%)	208 (19.8%)		
Normal weight (18.5 to <25 kg/m^2^)	536 (61.4%)	580 (55.2%)		
Overweight (25 to <30 kg/m^2^)	94 (10.8%)	174 (16.6%)		
Obese (>30 kg/m^2^)	31 (3.6%)	87 (8.3%)		

a Adjusted for age, sex, physical activity, tobacco use, education level, and socioeconomic status;

^b^Ordinal logistic regression used for all analyses of body mass index categories;

^c^Numbers do not add up due to missing data.

The bold values indicate statistical significance, *p*-value < 0.05.

**Figure 1:**
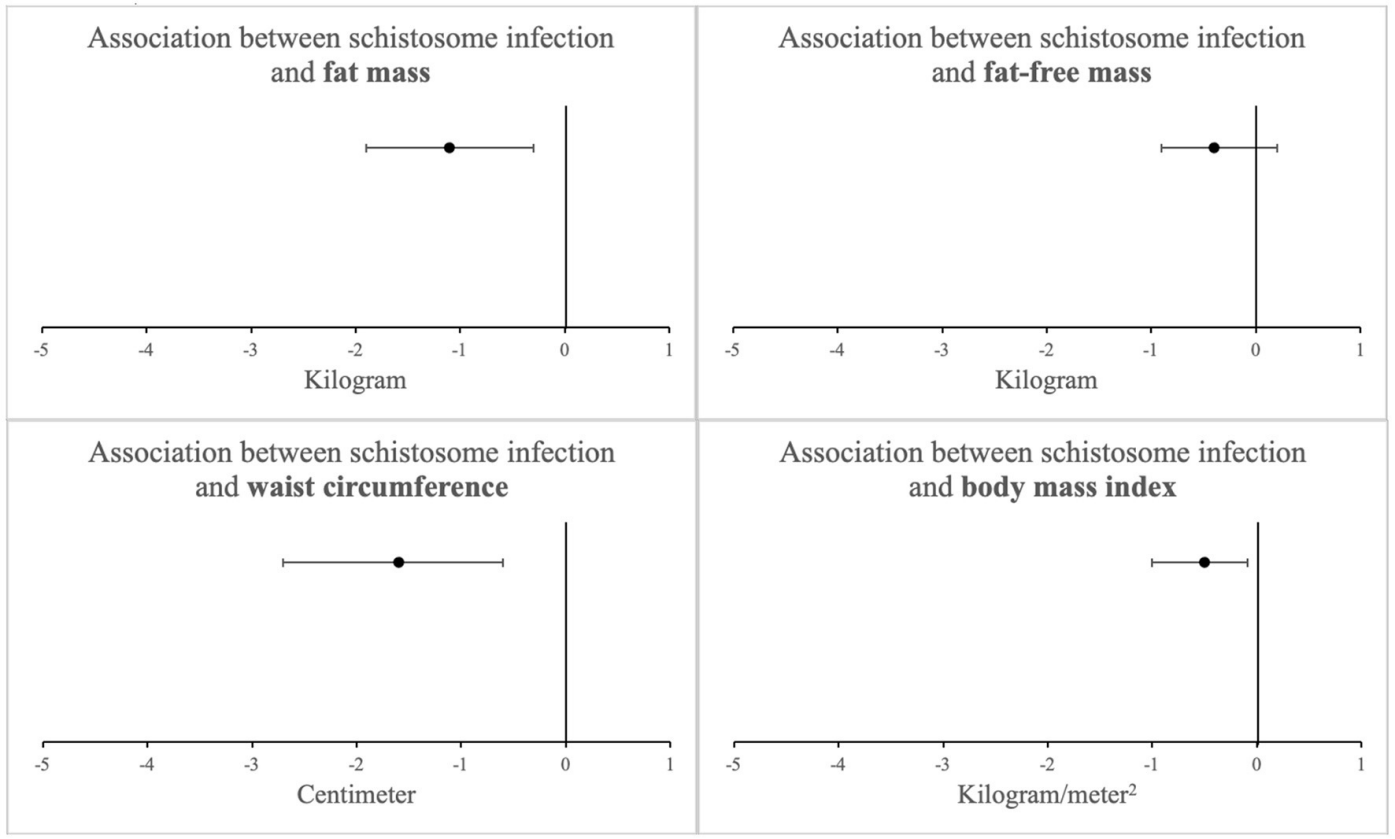
Association between schistosome infection and measures of adiposity among 1923 study participants after adjusting for potential confounders (linear regression coefficients with 95% confidence intervals)^a^. ^a^Adjusted for age, sex, physical activity, tobacco use, education level, and socioeconomic status, No significant interaction was observed by HIV status (see Table 3).

Adjusted regression models demonstrated that schistosome infection remained inversely associated with FM (adjusted regression coefficient: −1.1 kg [−1.9 to −0.3]; *p* = 0.009). All other continuous measures of adiposity also remained lower in schistosome-infected individuals after adjustment. Of note, FFM remained similar between the two groups (Figure 1). Further adjustments also showed that CAA level, a well-recognized proxy for *Schistosoma* worm burden (9), did not change the estimate of adiposity beyond the effect of being schistosome-infected.

Additionally, there were no interactions between HIV or ART and schistosome infection with respect to adiposity (*p* > 0.05) (Table 3).

**Table 3:** Effect modification by HIV status on the association between schistosome infection and measures of adiposity (interaction terms with 95% confidence intervals)^a^.

**Characteristic**	**HIV-infected ART-naïve^b^** **(interaction term [95% CI], *p*-value)**	**HIV-infected on ART^b^** **x(interaction term [95% CI], *p*-value)**
Fat mass (kg)	−0.75 [−2.4, 0.9], *p* = 0.39	0.48 [−1.7, 2.6], *p* = 0.66
Fat free mass (kg)	−0.06 [−1.3, 1.1], *p* = 0.93	0.20 [−1.3, 1.7], *p* = 0.80
Waist circumference (cm)	−0.11 [−2.3, 2.1], *p* = 0.92	0.88 [−1.8, 3.5], *p* = 0.52
Mid-upper arm circumference (cm)	−0.65 [−1.5, 0.2], *p* = 0.12	0.36 [−0.7, 1.4], *p* = 0.51
Body mass index (kg/m^2^)	−0.27 [−1.2, 0.6], *p* = 0.54	0.36 [−0.7, 1.5], *p* = 0.52

a Adjusted for age, sex, physical activity, tobacco use, education level, and socioeconomic status.

b Comparisons made to HIV-uninfected individuals.

## Discussion

It is well-known that schistosome infection, which is highly prevalent in sub-Saharan Africa, is associated with less adiposity and that less adiposity is associated with lower cardiovascular disease. We have demonstrated the importance of adjusting for socioeconomic and demographic variables in assessing the relationship between schistosome infection and cardiovascular disease. Adjusting for these variables explained approximately half of the difference in adiposity between schistosome-infected and uninfected study participants. These findings are consistent with experimental mouse models free of confounding, in which schistosome infection was associated with decreased body weight (12).

Even after adjustment, schistosome infection was independently associated with 1 kg lower FM with no effect on muscle mass. Our study confirms and extends prior human and animal studies (3, 4, 12) that have shown decreased BMI and other measures of body composition in people and mice with schistosome infections. Interestingly, higher loads of *Schistosoma* worm infection did not further magnify the association between infection and adiposity. This suggests that the weight loss associated with schistosome infection is unlike that of other chronic infectious diseases, such as HIV and tuberculosis, which cause wasting of both FM and FFM, and may be driven by alternative metabolic pathways (13, 14). Given known effects of obesity on cardiovascular disease (15), such reduction in adiposity could have an impact on cardiovascular health.

Although the majority of published data indicate a link between schistosome infection and lower adiposity, there are a few exceptions (16). Notably, this study differs from our previous study which used stool eggs to diagnose schistosomiasis (8), which likely underestimated the prevalence of schistosome infection and lowered the study's power to detect differences in adiposity. Antigen testing for schistosome infection has higher sensitivity than egg microscopy, particularly in women and HIV-infected individuals (17).

Our study has strengths and weaknesses. Strengths include a large, well-characterized cohort including many sociodemographic, clinical, and behavioral factors and use of antigens to detect schistosome infection with greater sensitivity. A cross-sectional design limits our ability to infer causality, warranting prospective data to assess the potential causal relationship between schistosome infection and adiposity. Such studies may also consider including cardiovascular outcomes to assess how schistosome infection and adiposity are interrelated and whether they impact non-communicable diseases. Additionally, those studies could consider examining how anthelmintic treatment and the duration of schistosome infection may impact cardiovascular outcomes cross-sectionally and longitudinally.

In conclusion, our study confirms that schistosome infection is associated with lower FM without reduction in FFM and that the potential protective effects of schistosome infection are not explained by confounders, co-infections, or the intensity of schistosome infection. Humans have lived with schistosomes for millennia, and it would therefore not be surprising if coevolution has resulted in beneficial cardiovascular effects of low-level schistosome infection on human health. Control of schistosomiasis globally has been based on large-scale treatment of at-risk population groups, improved sanitation and access to clean water, and control of snails that transmit the parasite (1). Although these policies have reduced the burden of schistosomiasis, infection is still quite prevalent in many tropical and subtropical regions, including Tanzania (1, 2). Our challenge is to identify and harness these beneficial effects while avoiding the harmful effects that result from high schistosome burden.

## Data availability statement

The datasets presented in this article are not readily available because data cannot be shared publicly but are available upon request and approval by the Medical Research Coordinating Committee (MRCC) of the National Institute for Medical Research (NIMR) in Tanzania, which requires that data should not be transferred or shared without their permission. For researchers who meet the criteria for access to confidential data, they may use the contact details below to request the data. Requests to access the datasets should be directed to The Secretariat, National Institute for Medical Research, 2448, Baraka Obama Road, P O Box 9653 Dar Es Salaam, Tanzania. ethics@nimr.or.tz.

## Author contributions

KP: writing—original draft, writing—review and editing, formal analysis, and visualization. GP: conceptualization, funding acquisition, methodology, project administration, validation, and writing—reviewing and editing. DF-J and MO: conceptualization, funding acquisition, methodology, and writing—reviewing and editing. BKa and BKi: project administration, validation, and writing—reviewing and editing. PC and CD: writing—review and editing and validation. HF and SF: conceptualization, funding acquisition, methodology, and writing—reviewing and editing. JD and RP: writing—review and editing, formal analysis, visualization, validation, and supervision. All authors contributed to the article and approved the submitted version.
